# Automated control for investigation of the insufflation-ventilation interaction in experimental laparoscopy

**DOI:** 10.1371/journal.pone.0285108

**Published:** 2023-05-05

**Authors:** Willem van Weteringen, Frank Sterke, John Vlot, René M. H. Wijnen, Jenny Dankelman

**Affiliations:** 1 Department of Pediatric Surgery, Erasmus MC Sophia Children’s Hospital, University Medical Center Rotterdam, Rotterdam, The Netherlands; 2 Department of BioMechanical Engineering, Faculty of Mechanical Engineering, Delft University of Technology, Delft, The Netherlands; University College Dublin - National University of Ireland: University College Dublin, IRELAND

## Abstract

In laparoscopic surgery the abdominal cavity is insufflated with pressurized carbon dioxide gas to create workspace. This pressure is exerted through the diaphragm onto the lungs, competing with ventilation and hampering it. In clinical practice the difficulty of optimizing this balance can lead to the application of harmfully high pressures. This study set out to create a research platform for the investigation of the complex interaction between insufflation and ventilation in an animal model. The research platform was constructed to incorporate insufflation, ventilation and relevant hemodynamic monitoring devices, controlling insufflation and ventilation from a central computer. The core of the applied methodology is the fixation of physiological parameters by applying closed-loop control of specific ventilation parameters. For accurate volumetric measurements the research platform can be used in a CT scanner. An algorithm was designed to keep blood carbon dioxide and oxygen values stable, minimizing the effect of fluctuations on vascular tone and hemodynamics. This design allowed stepwise adjustment of insufflation pressure to measure the effects on ventilation and circulation. A pilot experiment in a porcine model demonstrated adequate platform performance. The developed research platform and protocol automation have the potential to increase translatability and repeatability of animal experiments on the biomechanical interactions between insufflation and ventilation.

## Introduction

During laparoscopic surgery, pressurized carbon dioxide (CO_2_) gas is insufflated into the abdominal cavity. This gas volume forms the workspace in which the surgeon can operate. The pressure needed to create this workspace is exerted not only onto the abdominal wall, but onto the vascular system and internal abdominal organs as well [1]. Moreover, the intra-abdominal volume competes with the volume of the lungs through displacement of the diaphragm, causing a decrease in lung volume with the increase in intra-abdominal gas volume [2–4]. This impairs the ability to ventilate patients during laparoscopy, requiring an increase in intrapulmonary pressure to maintain sufficient inspiratory lung volume and gas exchange and to prevent collapse during expiration. However, increased intrapulmonary pressures can result in lung damage and prolonged recovery [5, 6].

Extensive literature exists on the negative consequences of high pressures applied in ventilation and insufflation in patients undergoing laparoscopic surgery [7–9]. Although several studies have investigated the interaction between insufflation and ventilation, these have not led to methods for optimization during surgery [10, 11]. Separate guidelines have been developed, advising minimization of mechanical ventilation (MV) and insufflation pressures [12, 13]. Investigation of the interaction between insufflation and ventilation is complicated by the fact that both are simultaneously affected by pressure changes on either side of the diaphragm.

An important effect of abdominal insufflation with CO_2_ gas is an increase in blood CO_2_ levels [14, 15]. This results from a reduction in lung volume and consequently impaired ventilation, as well as from CO_2_ uptake from the peritoneal cavity into the blood. In turn, high blood CO_2_ levels affect the muscle tone of blood vessel walls, affecting physiological parameters throughout the body [16–18]. Hence, control of the blood CO_2_ level is needed to reduce these effects. Management of oxygen (O_2_) is equally important, since even short periods of hypoxia can lead to a marked change in for example heart rate [19]. Other important factors influencing the interaction between insufflation and ventilation are compliances of the lungs and the abdomen, and the tone of the diaphragm and other muscles surrounding the abdominal and thoracic cavities. The muscle tone can be reduced using muscle relaxants, theoretically resulting in a more direct interaction between insufflation and ventilation [20–22].

The reproducibility of studies on biomechanics strongly depends on the methods for controlling parameters and on reducing the influence of variation between test subjects on outcome [23, 24]. A feasible method for investigating the interaction between ventilation and insufflation involves fixating the tidal lung volume while varying the insufflation pressure. This makes the abdominal volume and the ventilation pressure outcome parameters of changes in intra-abdominal pressure (IAP). The most accurate method for the repeated measurement of volumes is computed tomography (CT) imaging, which is only feasible in an animal model [25]. For investigation of the complex interactions involved, closed-loop control of ventilation parameters is very suitable for exclusion of the influence of blood gas fluctuations [26–28]. Although in clinical care closed-loop systems are increasingly used to stabilize patient conditions, in research such systems are rarely used to stabilize experimental conditions [29].

The aim of this study was to develop a research platform for investigation of the biomechanical interaction between surgical insufflation and mechanical ventilation in an animal model. To ensure repeatable conditions between experiments, the development of a closed-loop ventilation system aimed to minimize the effect of blood gas fluctuations. A pilot experiment was set up to evaluate the research platform and investigation method.

## Materials and methods

### Design considerations

Previous studies have shown that pig models are most representative of human physiology when investigating pulmonary dynamics and abdominal insufflation as separate topics [2, 30, 31]. The developed research platform had to be transportable and had to fit within the gantry of a CT scanner. To avoid accidental disconnection of data connections, vascular access or ventilation, the choice was made to create a single platform that houses the devices and animal. This provided several restrictions in the choice for the animal size and the number of devices. Animals with a weight approximating that of an adult human, in combination with the required devices, would not fit onto a CT slide and would exceed the maximum weight tolerance. To this end, the target weight of the animal model was set to 20 kg.

### Selection of devices

#### Insufflation and ventilation

The core of the research platform are an insufflation device and a mechanical ventilator (Table 1). Both devices can be read out and controlled through a serial connection, allowing remote and automated control over all insufflation and ventilation parameters. Remote control of the ventilator is required to shorten the apneic time during CT scanning breath holds. Automated control in the form of closed-loop control of ventilation parameters is needed to adjust blood gas levels. Due to size considerations of both the device and the animal model, a neonatal/pediatric ventilator was chosen. The following devices were chosen:

- Insufflation: Endoflator UI 40 (Karl Storz SE & Co. KG, Tüttlingen, Germany).
- Mechanical ventilation: Fabian HFO (Acutronic AG, Hirzel, Switzerland).
  In addition to the pressure and flow measurements provided by these devices, a high-resolution system was chosen to verify insufflator and mechanical ventilator measurements:
- To verify measurements of pressure and flow: heated pneumotachographs (PNT 8410A, Hans Rudolph Inc., Shawnee, KS, United States) combined with pressure sensors that also provided the option of connecting an esophageal balloon catheter for intrathoracic pressure measurements.

**Table 1:** Devices.

	Description	Device	Manufacturer
Control	Mechanical ventilator	fabian™ HFO	Acutronic Medical Systems AG
	Surgical insufflator	Endoflator^®^ 40 UI	Karl Storz GmbH
	Patient monitor	Root^®^	Masimo^®^ Corporation
Measurement	Pulse oximeter	Radical-7^®^ Pulse CO-oximeter	Masimo^®^ Corporation
	Patient monitor	IntelliVue MP20	Koninklijke Philips N.V.
	Blood pressure	Custom	Not applicable
	Electrocardiography	Custom	Not applicable
	Hemodynamic monitor	Pulsioflex™	Pulsion Medical Systems SE
	Hemodynamic monitor	PiCCO_2_™	Pulsion Medical Systems SE
	Syringe pump	Injectomat Agilia^®^	Fresenius Kabi AG
	Infusion pump	Infusomat^®^ Space^®^ P	B. Braun Melsungen AG
	Neuromuscular blockade	TOFscan^®^	Drägerwerk AG & Co. KGaA
	Trocar pressure/flow	Custom	Not applicable
	Transcutaneous blood gases	SDM / OxiVenT™ Sensor	Sentec AG
Infrastructure	Wifi router	Mi Wi-Fi Mini	Xiaomi
	Power backup unit	SMT1500IC	Schneider Electric Industries SAS
	Software	LabVIEW™ 2018	National Instruments Corp.
	Serial device hub (2x)	NPORT 5650-8-DT-J	MOXA Inc.
	Measurement/control computer	Universal: requires 3x USB and LabVIEW™ software	
	Remote control laptop	Universal: requires Microsoft Remote Desktop Protocol support	

List of selected devices, essential devices are required to control oxygenation and carbon dioxide levels and store data, other devices have been added to gather additional clinical and scientific data. Auxiliary devices have been added to overcome logistical challenges, for example, remote control during CT measurements.

#### CO_2_ and O_2_ monitoring

Monitoring devices were selected to observe the physiological effects of ventilation and insufflation. To increase the translational value of the measured parameters, standard of care devices were selected. All monitoring devices were required to have a connection for data readout. Commonly used technologies for CO_2_ and O_2_ monitoring were selected with response times and accuracy that would be able to provide input to a closed-loop ventilation algorithm. The following devices were selected:

- Monitoring the arterial oxygen saturation (SaO_2_): pulse oximetry (Masimo SET®, Masimo, Irvine, CA, United States) was used which provided a peripherally measured oxygen saturation (SpO_2_). For redundancy, three pulse oximeters were included, of which one with the Oxygen Reserve Index (ORI™). The ORI is calculated from arterial and venous saturation levels, which allows detection of oxygen levels surpassing full arterial hemoglobin saturation [32]. These sensors were connected to the mechanical ventilator, a Masimo ROOT and Masimo Radical 7 device.
- Measurement of cerebral and tissue oxygen levels: near-infrared spectroscopy (Masimo O3).
- Oxygen uptake: central venous oxygen saturation (ScvO_2_) measured with an intravascular optical catheter (CeVOX) connected to a monitor (PiCCO_2_, Getinge AB, Getinge, Sweden).
- End-tidal capnography (etCO_2_) was chosen as the primary CO_2_ measurement, providing a continuous measurement with good accuracy during pneumoperitoneum [5, 33]. A mainstream capnograph (Philips Capnostat 5, Philips, Eindhoven, The Netherlands) was selected that could be interfaced directly with the mechanical ventilator.
- For non-invasive monitoring of the arterial partial pressures of carbon dioxide (PaCO_2_) and oxygen (PaO_2_), transcutaneous blood gas sensor (Sentec OxiVenT, Sentec AG, Therwil, Switzerland).

#### Circulation monitoring

The primary circulatory parameters that needed to be measured were heart rate, blood pressure and cardiac output.

- Electrocardiography: a compact patient monitor with optional filtering for electric interference (Philips MP40, Philips, Eindhoven, The Netherlands).
- Arterial and venous blood pressures were recorded at a sampling rate of 1000 Hz for offline pulse contour analyses. Pressures were measured using disposable transducers (Meritrans DTX Plus, Merit Medical Ireland Ltd, Galway, Ireland) combined with two pre-amplifiers (CPJ2S, SCAIME SAS, Juvigny, France) and an analog-to-digital converter (USB-6002 DAQ, National Instruments, Austin, Texas, United States).
- For cardiac output (CO) measurements, the gold standard involves placement of a Swan-Ganz catheter within the heart. As the invasiveness of this method is poorly tolerated by the 20 kg porcine model, an alternative method was chosen. An intermittent absolute CO measurement was combined with a continuous relative CO measurement. This combination allows calibration of the pulse contour measurement at the beginning of an experiment. The absolute CO was measured with cold fluid thermodilution (PiCCO_2_ monitor). Continuous CO was monitored with pressure-based pulse contour analysis (ProAQT sensor and PulsioFlex monitor, Getinge AB, Getinge, Sweden). The catheter for the fluid thermodilution measurement had a temperature sensor at the tip, which provided a continuous core temperature measurement.

#### Anesthesia, sedation and muscle relaxation

Monitoring of anesthetics, sedatives and fluids was included, as it was considered essential in maintaining hemodynamic stability and minimizing differences between experiments.

- Infusion and fluid management: pumps with a serial data output (Braun Infusomat® Space, B. Braun, Melsungen, Germany and Fresenius Kabi Injectomat® MC Agilia, Fresenius Health Care Group, Hamburg, Germany).
- To monitor the effect of neuromuscular blockade (NMB) and titrate the administered dose over time: train-of-four (TOF) and post-tetanic count (PTC) monitoring. The frequency of TOF and PTC measurements is limited due to the temporary and local depletion of neurotransmitters by the tetanic stimulus. To allow both measurements continuously and simultaneously two devices were included for placement at different extremities (Dräger TOFscan®, Drägerwerk AG & Co. KGaA, Lübeck, Germany).

### Data acquisition and protocol management

Serial data communication between all devices and a central computer system allows time-synchronized collection of all data streams, as well as control over insufflation and ventilation whilst running the experimental protocol. The central computer runs the program for managing the interface and closed-loop controller. The program was created using software for programming that included support for interface development, serial communication and automated file naming based on the study protocol and measurements, as well as timestamping of files and measurements (LabVIEW 2018 SP1, National Instruments, Austin, Texas, United States). A protocol management system was programmed that could execute the pre-programmed experimental steps and monitor progress. Timers and protocol step control were implemented in the interface. Protocol deviations could be corrected using manual overrides.

### Closed-loop ventilation and oxygenation

The aim of the closed-loop controller is to separate the control of O_2_ levels from the control of CO_2_ levels during insufflation by adapting MV settings. To achieve this, a set of possible system inputs (e.g. MV settings) and outputs (levels of CO_2_ or O_2_) was evaluated. Since all insufflators are pressure-controlled, IAP was selected as the controlled parameter for creating the surgical workspace. To prevent lung collapse and impairment of ventilation with increasing IAP, tidal volume-controlled MV was preferred over pressure-controlled MV. Other MV parameters that can be controlled are the fraction of inspired oxygen (FiO2), tidal volume (Vt), positive end-expiratory pressure (PEEP), inspiratory-expiratory time ratio (I:E-ratio) and respiratory rate (RR). These parameters were evaluated for three criteria:

1. Adapting the parameters should have limited impact onto the investigated insufflation-ventilation interaction.
2. Adapting the parameter should control the level of O_2_ or CO_2_.
3. O_2_ and CO_2_ levels should be controllable separately, with minimal influence on the other parameter.

For controlling oxygen levels, FiO_2_ was the only candidate system input. For stabilizing CO_2_ levels RR, Vt, PEEP and the I:E ratio were considered. RR was selected as system input, mainly because Vt and PEEP have a stronger impact on the investigated pressure-volume relationship and changing the I:E ratio was expected to provide limited control over CO_2_ levels.

Any CO_2_ or O_2_ measurement that could be obtained was considered as a potential input parameter. This included the transcutaneously measured partial pressure of carbon dioxide (tcPCO_2_), etCO_2_, SaO_2_, and ORI measurements. These measurements were evaluated for their accuracy, sensitivity in identifying changes in O_2_ or CO_2_ levels, as well as clinical usability. Oxygen levels were monitored using both SpO_2_ and ORI, to allow control over PaO_2_ levels in the range where hemoglobin saturation and subsequent SpO_2_ values have reached 99 to 100%. SpO_2_. For management of CO_2_ levels, etCO_2_ was chosen as the output parameter. Although a measurement can be accurate, there is a delay between changing the MV settings and its effect. Changing the MV settings before observing the effect from the previous change could lead to swings in O_2_ and CO_2_ levels. The minimal time interval at which these effects are observed can be translated to a maximal control rate at which the closed-loop controller is executed. Response times and control stability were based on the physiology of the selected animal model and set to a 40 second interval.

### Pilot experiment

#### Subject

The performance of the closed-loop ventilation system, selected measurements, data acquisition and protocol management system were evaluated in a pilot experiment. A female Landrace pig with a targeted weight of 20 kg was selected for this pilot experiment. Environmentally enriched housing was provided. Until the start of the experiment water was available to the animal ad libitum, food was available until the morning of the experiment.

#### Ethics statement

All samples and data were collected using procedures in accordance with the Dutch Animal Testing act. The license number for this study for the Central Authority for Scientific Procedures on Animals was AVD101002015180. Institutional approval was given by the Animal Ethics Committee, protocol number 15-180-02,2,1. All experimental steps were executed according to pre-approved standard operating procedures.

#### Anesthesia and instrumentation

Before premedication of the animal, all devices were time-synchronized and pressure and flow measurements were calibrated. Intramuscular premedication was administered to the animal with ketamine 30 mg/kg, midazolam 1 mg/kg and atropine 0.03 mg/kg, followed by a 15 minute time window for the sedation to take effect. Adequate sedation was confirmed with nociceptive stimuli and by the absence of the corneal reflex. The animal was placed in supine position and after application of lidocaine onto the vocal cords it was intubated with a cuffed endotracheal tube. An esophageal balloon catheter was then placed. For maintenance of anesthesia an auricular intravenous cannula was placed, through which propofol 14 mg/kg/h and sufentanil 6.5 mg/kg/h were administered continuously. Fluid maintenance was provided with a crystalloid solution. Three-lead electrocardiography (ECG) monitoring was started with an electrode configuration tailored to pigs [34]. Using the modified Seldinger technique, intravascular access was obtained; a catheter in the femoral artery, a PiCCO catheter in the other femoral artery, a sheet in the femoral vein, and a multi-lumen catheter in the jugular vein in which a CeVOX catheter was placed. Near-infrared spectroscopy (NIRS) oxygen monitoring was placed over the brain and on the shoulder. A transcutaneous blood gas sensor was attached to the neck. A bladder catheter was placed. At the supra-umbilical level, a 12 mm trocar was placed (VersaOne™ Bladeless Optical Trocar with Fixation Cannula, Medtronic, Fridley, United States). Intraperitoneal placement was verified endoscopically.

#### Muscle relaxation

To control the effect of muscle tension on the interaction between intra-abdominal and intrathoracic pressures muscle relaxation was applied. For this pilot experiment deep muscle relaxation was chosen to exclude any effects of diaphragmatic muscle tension. The NMB monitors were attached to both lower limbs. Rocuronium levels were titrated to a target TOF of 0 with a 50 mA stimulus and a PTC value of less than 2. Infusion of rocuronium was provided both cranially and caudally to minimize any blood pooling and release effects due to compression of abdominal blood vessels during abdominal insufflation and exsufflation.

#### Study protocol

After induction of anesthesia, instrumentation and titration of muscle relaxation, the research platform was transported from the lab to the CT scanner, where the study protocol was started. Ventilation was closed-loop controlled with a volume guarantee of 7.5 ml/kg and a PEEP of 5.0 hPa. To maintain stable CO_2_ levels mild hypercapnia was permitted with a target etCO2 of 7.0 kPa. The minimum allowed SpO_2_ level was set to 97%, the ORI target range was set to 0.0–0.4. After the CO_2_ insufflator had been attached to the trocar, insufflation was started and the abdominal insufflation was applied at pressure levels of 0, 5, 8, 10, 12, 14, 16, 18, 20, 16, 10, 5 and 0 hPa in a stepwise fashion. A stabilization time of 3 minutes was implemented between each step, after which a CT scan was made during an expiratory and inspiratory breath hold. After the experiment the animal was terminated under general anesthesia with a bolus of 10 ml potassium chloride 10%. The animal’s organs were inspected for abnormalities at necropsy.

#### Measurements and analysis

To evaluate performance of the measurement devices, the closed-loop ventilation system and the protocol management system parameters on insufflation, oxygenation, ventilation and hemodynamics were plotted over time. Data was processed in MATLAB R2021a (The MathWorks, Inc., Natick, MA, United States) and visualized using Prism 9.2.0 (GraphPad Software, San Diego, CA, United States).

## Results

### Research platform

**Fig 1:**
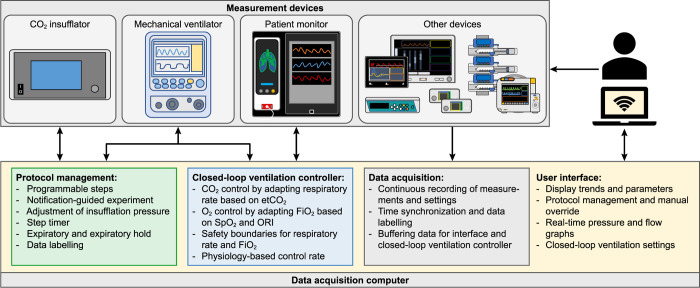
Research platform communication diagram. The primary measurement devices were the CO_2_ insufflator, mechanical ventilator and patient monitor, which were coupled to the protocol management system and the closed-loop ventilation controller. The other monitoring devices were additionally recorded continuously. The main computer had four main functions, which included protocol management, closed-loop ventilation control, data acquisition and the presentation of the user interface. The researcher had the option to manually control all devices, as well as override the protocol and closed-loop system when needed.

A functional and connectivity layout was designed for the research platform (Fig 1). The control software program was designed in separate modules which managed data acquisition and storage, protocol control, the interface, closed-loop ventilation and remote control. The separation of the modules minimized interdependency, allowing individual re-initialization of modules in case of failure during the experiment. The devices and central computer were mounted onto a X-ray translucent slide that, together with the animal, could be placed in a CT scanner. The entire platform could be transported on a cart that housed the insufflator, infusion pumps and a power backup unit.

### Interface and protocol management

An interface was created incorporating all functionalities of the research platform. For monitoring the condition of the subject, actual numbers and trends of vital parameters were shown. This was complemented by the settings and readings from the fluid pumps, insufflator and ventilator. Graphs of the real-time high-resolution pressure and flow measurements were implemented for monitoring of the pressure-volume relation. Settings for closed-loop ventilation control were provided, as well as manual control. Protocol management consisted of pre-programmed experiment steps that could be executed, automatically labelling all acquired data accordingly. This included storage of all devices settings.

### Closed-loop mechanical ventilation

#### Algorithm

**Fig 2:**
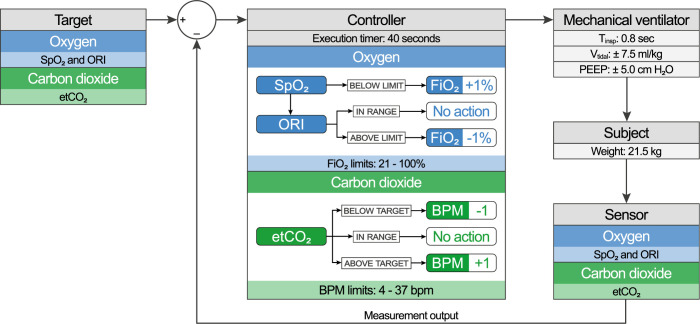
Automated ventilation control diagram. Targets for O_2_ and CO_2_ levels were compared to the measured saturation, ORI and etCO_2_. The controller algorithm used SpO_2_ and ORI values to adjust FiO_2_ and etCO_2_ to adjust the respiratory rate. Tidal volume guarantee and inspiration time were set to a fixed value.

CO_2_ and O_2_ levels were separately controlled by an algorithm (Fig 2). Target ranges of SpO_2_, ORI and etCO_2_ could be input to the controller. Based on the provided continuous measurements, the controller determined the commands to be sent to the mechanical ventilator within the provided restraints. To provide comparable information on response times, the execution interval of 40 seconds was kept constant. Actuation was halted during breath holds. Measurements were checked for validity, the most recent 5 seconds of data were averaged and input to the algorithm.

#### Oxygenation

The inspired fraction of oxygen (FiO_2_) was automatically adjusted to control SpO_2_ and ORI levels, with the aim of providing an oxygen buffer for the breath holds by increasing the FiO_2_ with 1% when in an SpO_2_ range of 97–98% and an ORI range of 0.0 to 0.4. At a higher ORI level FiO_2_ was decreased with steps of 1%.

#### Carbon dioxide ventilation

To minimize the effect ventilation adjustments have onto static and dynamic pulmonary and abdominal mechanics, the tidal volume was fixed using volume guarantee. To prevent dynamic changes from affecting the lung condition over time, the inspiratory time was fixed at 0.8 s. This allowed automated adjustment of the RR in a range of 4 to 37 bpm to achieve the CO_2_ target. For safety, the RR could only adapt one bpm up or down every 40 seconds with the lower RR limit set to 10 bpm. To accommodate the expected increase in CO_2_ load during insufflation, permissive hypercapnia was applied with an etCO_2_ target of 7.0 kPa.

### Platform evaluation

**Fig 3:**
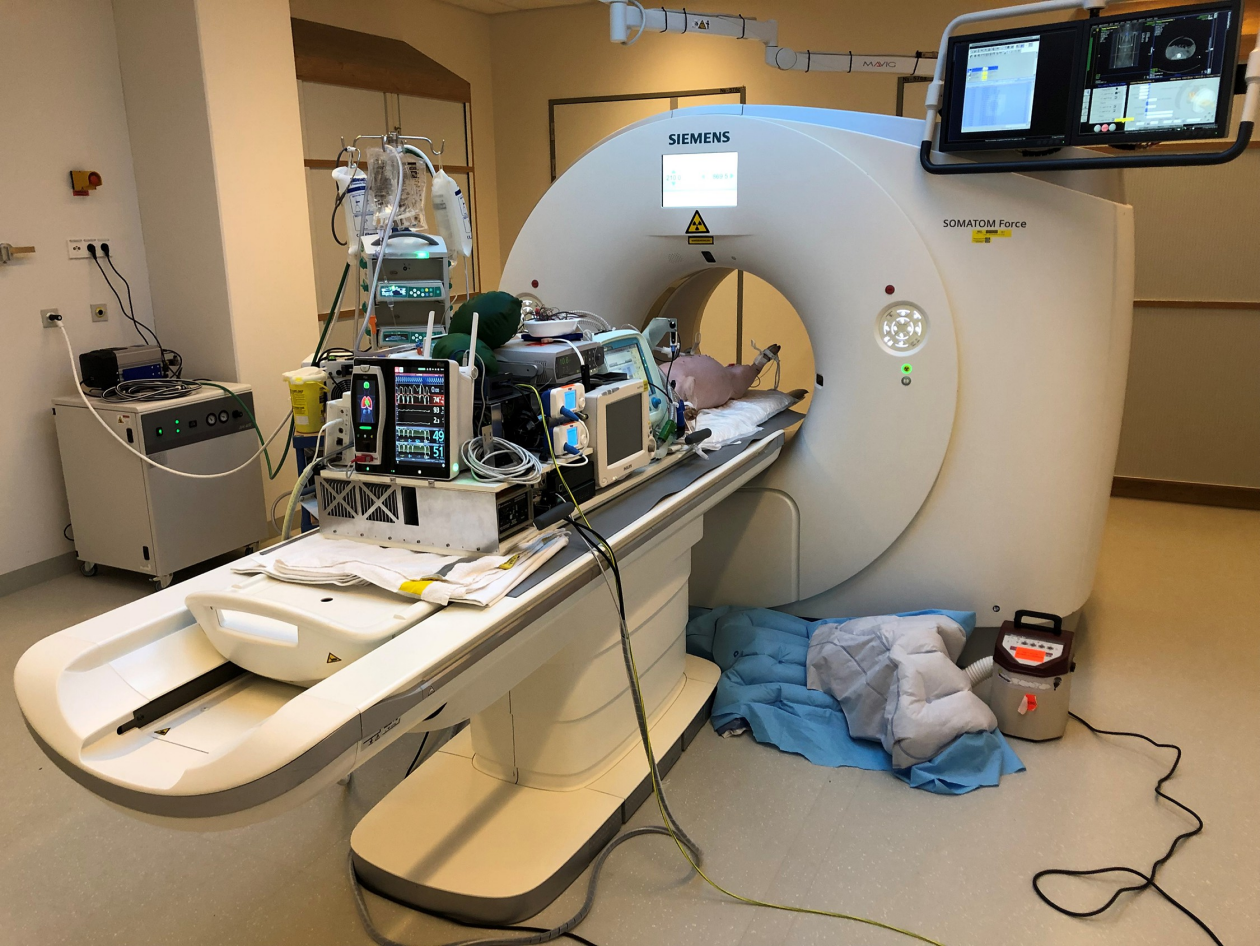
Experimental platform. Experimental platform during the pilot experiment with CT measurements in a porcine animal model.

**Fig 4:**
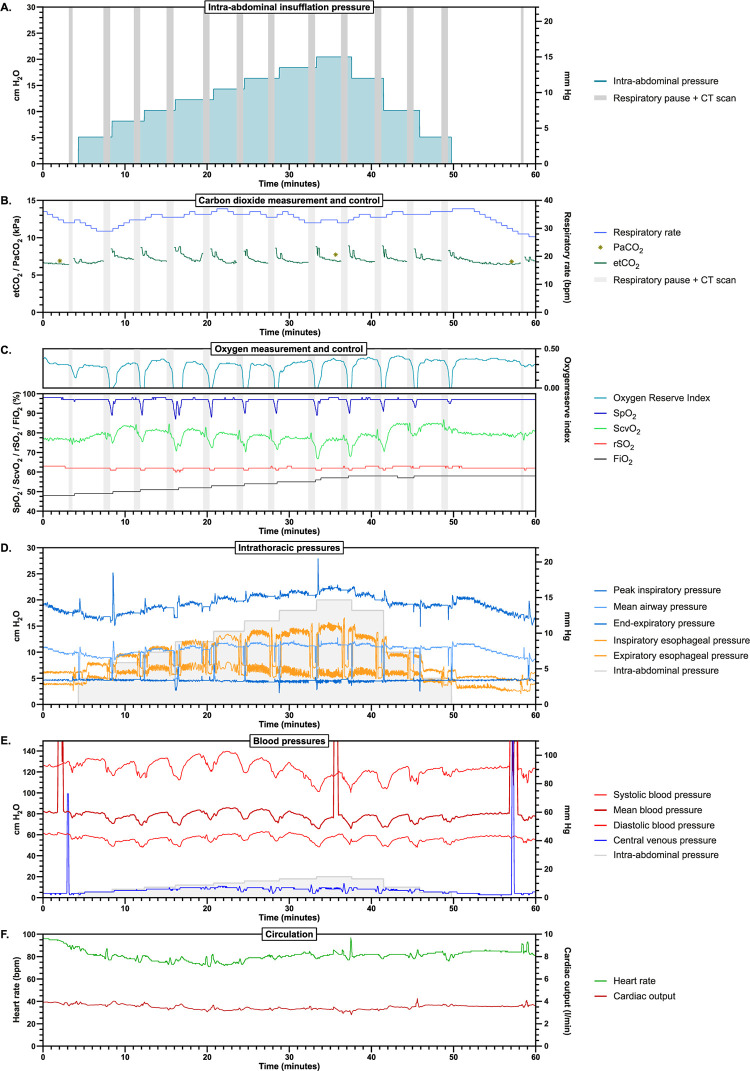
Physiological measurements from pilot experiment. The grey vertical bars indicate the respiratory pause for CT scanning. (A) Intra-abdominal insufflation pressure settings over time. (B) Measurement and control of CO_2_. The closed-loop adjusted respiratory rate setting of the mechanical ventilator in breaths per minute (blue line, right y-axis). End-tidal CO_2_ levels measured at the airway opening (green line, left y-axis). The asterisks indicate the PaCO_2_ measured from blood sampling. (C) Measurement and control of O_2_. The oxygen reserve index (green line, right axis). Oxygen saturation levels at different locations: SpO_2_, ScvO_2_ and rSO_2_ (%, blue, green and red lines, left axis). The automatically adjusted FiO_2_ provided by the mechanical ventilator (%, black line, left axis). (D) Intrathoracic pressures during insufflation, shown in cm H_2_O on left y-axis, mm Hg on right y-axis. For reference the set insufflation pressures are shown (grey). Peak inspiratory, mean and end-expiratory airway pressures (blue lines), with the end-expiratory pressure set to 5 cm H_2_O / 3.75 mm Hg. Peak inspiratory and end-expiratory pressures measured in the esophagus (orange lines). (E) Blood pressures, shown in cm H_2_O on left y-axis, mm Hg on right y-axis. Arterial blood pressures; systolic, mean and diastolic (red lines). Central venous blood pressure (blue line). Insufflation pressures are shown as reference (grey). The pressure spikes are artefacts due to closing of the line to the pressure transducer for blood gas sampling. (F) Circulation parameters; heart rate (bpm, green line, left axis) and cardiac output (L/min, red line, left axis).

The platform was evaluated in a pilot experiment on a 21.5 kg pig. The mechanical ventilator’s volume guarantee mode was set to provide 160 mL per breath. Fig 3 shows the experimental platform during the experiment. The interaction between insufflation and ventilation and the resulting effects on other cardiorespiratory measurements during a series of increasing and decreasing IAP’s is shown in Fig 4. The closed-loop ventilation algorithm approximated etCO_2_ levels to 7.0 kPa after each IAP step within the set 3 minutes of stabilization time, maintaining variability of the respiratory rate. Maintenance of an oxygen buffer prevented low saturation levels following breath holds. Both arterial and venous blood pressures were markedly affected by the IAP steps and the breath holds, while heart rate and cardiac output remained unaffected.

## Discussion

A research platform was developed for investigation of the interaction between intra-abdominal surgical gas insufflation and mechanical ventilation in an animal model. Pressures and volumes of insufflation and ventilation were measured with sensors and CT scanning. Closed-loop control adjusted mechanical ventilation to target preset blood gas levels. A central computer system provided connectivity with all devices and enabled presentation of an interface, execution of the closed-loop ventilation controller, protocol management and automation of data acquisition.

This is the first method for the detailed investigation of the direct interaction between insufflation and ventilation through the control of IAP. Key in this method is the fact that insufflation and ventilation can be controlled through either pressure or flow. Volume control is in practice based on the integration of flow. Since no available insufflators provide flow control, there was the necessity to use pressure as the input parameter. The advantage of this method is that it allows the application of small pressure increments to investigate the resulting intra-abdominal volume. With only the diaphragm separating the thoracic and abdominal cavities, the direct pressure interaction during insufflation is ideally studied without mechanical ventilation. However, in practice this will lead to a decrease in lung volume and subsequently in tidal volume, which can only be countered using a mechanical ventilator that guarantees a set tidal volume. In addition, the investigation of the intra-abdominal insufflated gas volume resulting from the insufflation pressure is preferably not affected by lung collapse. For these reasons, the choice was made to use mechanical ventilation with volume guarantee. For investigations of lung dynamics one could prefer the use of pressure control ventilation, for which adaptation of the closed-loop ventilation controller is required. Although the PEEP was kept constant, most likely the residual lung volume was slightly decreased under the influence of insufflation pressures. Without the ability to measure and control residual lung volume, the use of a constant PEEP was preferred to keep experimental conditions constant. Combined with volume guarantee and a fixed inspiration time, changes in lung compliances could therefore be directly related to changes in the peak inspiratory pressure (PIP), as shown in Fig 4D.

This research platform is focused on controlling insufflation and mechanical ventilation, with the ultimate aim of optimizing patient conditions. The cardiovascular system, however, poses restrictions to the insufflation and ventilation pressures that can be applied. Despite the fact that perfusion outside of the thoracic and abdominal cavities is not easily affected due to the height of arterial blood pressures, venous return can already be affected by low pressures [35]. Perfusion of intra-abdominal organs such as the kidneys has been shown to be impaired during insufflation, most likely due to a strong decrease in venous return [36, 37]. In the thoracic cavity, similar impairment occurs when the pulmonary capillary wedge pressure is exceeded. However, pulmonary perfusion is affected by PEEP, PIP and the expiratory time. Investigation of insufflation and ventilation pressures should therefore always consider the effects on hemodynamics and tissue perfusion. For this reason the presented research platform includes continuous arterial and venous blood pressures measurements, as well as heart rate and cardiac output. The pilot experiment showed a stable central circulation, but organ perfusion parameters were not included. Although organ perfusion parameters would be very interesting for the investigation, the required perfusion scans using nephrotoxic contrast agents and placement of perfusion sensors that require incision of the abdominal wall were likely to have affected the investigation.

Systems for automated control of ventilation have been developed over the past 30 years [38]. The initial implementation in clinical devices has become common for functions that are based on mechanical feedback, such as volume control or pressure control options. Closed-loop systems in which the cardiorespiratory physiology is part of the loop are more challenging in terms of controllability due to longer or slower feedback loops, and are less predictable. As a consequence, clinical implementations have focused on specific single-parameter functions such as oxygen control [39]. Complex control systems are needed to deal with the variety in responses during clinical care. For investigation of the interaction between insufflation and ventilation a simple controller was chosen that was fast enough to compensate the relatively slow changes and corresponding physiological responses. The main challenge for this controller was to compensate for the respiratory pauses that were needed to minimize movement artefacts during CT scanning. Returning to the target levels within the set scanning interval of 3 minutes was therefore essential. Contrary to other controllers, the aim of this platform did not permit variation in tidal volumes, restricting the variable parameters to the ventilation frequency.

Using the volume of expired CO_2_ to investigate peritoneal CO_2_ uptake requires the etCO_2_ to be kept constant to rule out the effects of CO_2_ accumulation. Previous studies suggested that peritoneal CO_2_ uptake gradually increases because etCO_2_ levels and expired CO_2_ volumes increase continuously for several hours during laparoscopy [14, 15]. This results from the assumption that keeping ventilation parameters constant will provide PaCO_2_ or etCO_2_ as an indicator of CO_2_ load. Unfortunately, lung compliance changes during laparoscopy. If the mechanical ventilation parameters are not adapted, CO_2_ accumulates in the bloodstream and tissues. The accumulation process decouples the relation between peritoneal CO_2_ uptake and the expired volume of CO_2_. Increased etCO_2_ levels indicate an increase in PaCO_2_, which is a consequence of CO_2_ accumulation. Changes in lung compliance that occur during laparoscopy should therefore be addressed by adapting mechanical ventilation to keep etCO_2_ constant. The expired CO_2_ volume then approximates the net CO_2_ output. This is a sum of the metabolic CO_2_ production and peritoneal uptake. If the metabolic CO_2_ production is constant, the changes in expired CO_2_ volume reflect the changes in peritoneal CO_2_ uptake. Factors that affect the metabolic rate, such as muscle relaxation and anesthesia, should not be adjusted during the experiment.

The ORI has been introduced as a parameter to warn for impending hypoxia during induction of anesthesia and intubation procedures. Rapidly decreasing hyperoxia is masked during these procedures by the inability to measure the hyperoxic buffer while showing fully saturated hemoglobin. Maintenance of a hyperoxic buffer could be attractive in procedures in which regular respiratory holds are either needed or expected. Based on Fick’s principle, the index remains comparable over time as long as cardiac output and oxygen consumption are constant [32]. In the experimental setting ORI proved effective in achieving controlled hyperoxia, but in clinical practice the dynamic changes due to anesthesia and interventions might limit the use of this parameter. Regardless of the inaccuracy that can be introduced, it is likely that with the ORI parameter it will be possible to identify severe hyperoxia and adjust FiO_2_ accordingly.

With complex interactions, the degree of control of experimental conditions determines the relevance of output parameters. In the case of the presented research platform, lung mechanics and blood gas levels were identified as factors that should be controlled. If such indirect interactions are left uncontrolled, they considerably affect outcome and impair the reproducibility of experiments. In addition, the repeatability of results within experiments is increased as long as parameters are kept in check by closed-loop control. In our pilot experiment this was demonstrated by the return of CO_2_ and O_2_ values to the defined targets before each CT measurement. Exclusion of the vasotonic and cardiotonic influence of these factors increases comparability of heart rate, blood pressure and cardiac output measurements at these points in time. Similarly, by keeping Vt, PEEP and the inspiratory time constant they did not affect the PIP required for the target Vt, making the PIP and intra-abdominal volume direct interaction outcome parameters for the applied IAP.

During the pilot experiment, only the level of IAP was adapted to investigate its effect onto PIP, intra-abdominal volume, CO, HR and BP. In future studies, this platform could be used to accurately investigate factors influencing the interaction between insufflation and ventilation, such as the depth of muscle and diaphragm relaxation, the effect of body size and other inter-individual differences in tissue compliance. The clinical consequences of surgical insufflation are substantial, and mostly related to the applied pressure and interaction with ventilation. Amongst the most important topics that can be investigated using this research platform are the effects of insufflation on venous return and organ perfusion [40], the optimal ventilation strategy during minimal access surgery [41], and the benefits of personalized insufflation pressure [42]. Other potential applications include research on ventilation methods, for which other animal models can be considered [43]. The research platform, animal model and experimental procedure have been selected to minimize the translational gap to clinical care. With the exception of repeated CT scanning all measurements can be reasonably applied in a clinical setting. Closed-loop control could therefore be used in a similar manner to increase the reproducibility of outcome in clinical investigations.

## Conclusions

- The developed research platform enables the investigation of the interaction between insufflation and ventilation in a porcine model.
- Closed-loop ventilation is able to maintain CO_2_ and O_2_ at target values in a model for laparoscopic CO_2_ insufflation.
- Automated control of experiment protocol, insufflation and ventilation enhances reproducibility of in vivo studies and minimizes the translational gap between animal research and clinical application.

## Supporting information

S1 Checklist The ARRIVE guidelines 2.0: Author checklist. (PDF)

S1 Data (XLSX)

## Abbreviations

CO: Cardiac output
CO_2_: Carbon dioxide
CT: Computed Tomography
ECG: Electrocardiogram
etCO_2_: End-tidal partial pressure of carbon dioxide
FiO_2_: Fraction of inspired oxygen
IAP: Intra-abdominal pressure
I:E ratio: Inspiratory-expiratory time ratio
MV: Mechanical ventilation
NIRS: Near-infrared spectroscopy
NMB: Neuromuscular blockade
O_2_: Oxygen
ORI: Oxygen Reserve Index
PaCO_2_: Arterial partial pressure of carbon dioxide
PaO_2_: Arterial partial pressure of oxygen
PEEP: Positive end-expiratory pressure
PIP: Positive inspiratory pressure
PTC: Post-tetanic count
RR: Respiratory rate
SaO_2_: Arterial oxygen saturation
SpO_2_: Peripherally measured arterial oxygen saturation
ScvO_2_: Central venous oxygen saturation
TOF: Train of four
Vt: Tidal volume

## References

[1] Özdemir-van Brunschot DMD, van Laarhoven KCJHM, Scheffer GJ, Pouwels S, Wever KE, Warlé MC. What is the evidence for the use of low-pressure pneumoperitoneum? A systematic review. Surg Endosc. 2016;30: 2049–2065. doi: 10.1007/s00464-015-4454-9 26275545 PMC4848341

[2] Regli A, De Keulenaer BL, Singh B, Hockings LE, Noffsinger B, van Heerden PV. The respiratory pressure—abdominal volume curve in a porcine model. Intensive Care Med Exp. 2017;5. doi: 10.1186/s40635-017-0124-7 28243924 PMC5328886

[3] Loring SH, Behazin N, Novero A, Novack V, Jones SB, O’Donnell CR, et al. Respiratory mechanical effects of surgical pneumoperitoneum in humans. J Appl Physiol. 2014;117: 1074–1079. doi: 10.1152/japplphysiol.00552.2014 25213641 PMC4217051

[4] Malbrain MLNG, Peeters Y, Wise R. The neglected role of abdominal compliance in organ-organ interactions. Critical Care. Crit Care; 2016. doi: 10.1186/s13054-016-1220-x 26983963 PMC4794911

[5] Valenza F, Chevallard G, Fossali T, Salice V, Pizzocri M, Gattinoni L. Management of mechanical ventilation during laparoscopic surgery. In: Best Practice and Research: Clinical Anaesthesiology. 2010 pp. 227–241. doi: 10.1016/j.bpa.2010.02.002 20608559

[6] Futier E, Constantin JM, Jaber S. Protective lung ventilation in operating room: A systematic review. Minerva Anestesiol. 2014;80: 726–735. 24226493

[7] Jo YY, Kwak HJ. What is the proper ventilation strategy during laparoscopic surgery? Korean Journal of Anesthesiology. Korean Society of Anesthesiologists; 2017. pp. 596–600. doi: 10.4097/kjae.2017.70.6.596 29225741 PMC5716816

[8] Nguyen NT, Anderson JT, Budd M, Fleming NW, Ho HS, Jahr J, et al. Effects of pneumoperitoneum on intraoperative pulmonary mechanics and gas exchange during laparoscopic gastric bypass. Surg Endosc Other Interv Tech. 2004;18: 64–71. doi: 10.1007/s00464-002-8786-x 14625752

[9] Umano GR, Delehaye G, Noviello C, Papparella A. The “dark Side” of Pneumoperitoneum and Laparoscopy. Minimally Invasive Surgery. Hindawi Limited; 2021. doi: 10.1155/2021/5564745 34094598 PMC8163537

[10] Wauters J, Claus P, Brosens N, McLaughlin M, Hermans G, Malbrain M, et al. Relationship between abdominal pressure, pulmonary compliance, and cardiac preload in a porcine model. Crit Care Res Pract. 2012;2012. doi: 10.1155/2012/763181 22454767 PMC3290811

[11] Bloomfield GL, Ridings PC, Blocher CR, Marmarou A, Sugerman HJ. A proposed relationship between increased intra-abdominal, intrathoracic, and intracranial pressure. Crit Care Med. 1997;25: 496–503. doi: 10.1097/00003246-199703000-00020 9118668

[12] Moberg AC, Montgomery A. Pneumoperitoneum—Update 2006. EAES Guidelines for Endoscopic Surgery: Twelve Years Evidence-Based Surgery in Europe. Springer, Berlin, Heidelberg; 2006. pp. 87–95. doi: 10.1007/978-3-540-32784-4_3

[13] Neudecker J, Sauerland S, Neugebauer EAM, Bergamaschi R, Bonjer HJ, Cuschieri A, et al. The EAES clinical practice guidelines on the pneumoperitoneum for laparoscopic surgery (2002). EAES Guidelines for Endoscopic Surgery: Twelve Years Evidence-Based Surgery in Europe. Springer, Berlin, Heidelberg; 2006. pp. 39–85. doi: 10.1007/978-3-540-32784-4_2

[14] Eaton S, McHoney M, Giacomello L, Pacilli M, Bishay M, De Coppi P, et al. Carbon dioxide absorption and elimination in breath during minimally invasive surgery. J Breath Res. 2009;3. doi: 10.1088/1752-7155/3/4/047005 21386202

[15] McHoney M, Corizia L, Eaton S, Kiely EM, Drake DP, Tan HL, et al. Carbon dioxide elimination during laparoscopy in children is age dependent. Journal of Pediatric Surgery. J Pediatr Surg; 2003. pp. 105–110. doi: 10.1053/jpsu.2003.50021 12592630

[16] Nguyen NT, Wolfe BM. The physiologic effects of pneumoperitoneum in the morbidly obese. Annals of Surgery. Ann Surg; 2005. pp. 219–226. doi: 10.1097/01.sla.0000151791.93571.70 PMC1356906 15650630

[17] Gutt CN, Oniu T, Mehrabi A, Schemmer P, Kashfi A, Kraus T, et al. Circulatory and respiratory complications of carbon dioxide insufflation. Digestive Surgery. Dig Surg; 2004. pp. 95–105. doi: 10.1159/000077038 15010588

[18] Ho HS, Saunders CJ, Gunther RA, Wolfe BM. Effector of hemodynamics during laparoscopy: Co2 absorption or intra-abdominal pressure? J Surg Res. 1995;59: 497–503. doi: 10.1006/jsre.1995.1198 7564324

[19] Pingitore A, Gemignani A, Menicucci D, Di Bella G, De Marchi D, Passera M, et al. Cardiovascular response to acute hypoxemia induced by prolonged breath holding in air. Am J Physiol—Hear Circ Physiol. 2008;294. doi: 10.1152/ajpheart.00607.2007 17993602

[20] Bruintjes MH, Van Helden E V., Braat AE, Dahan A, Scheffer GJ, Van Laarhoven CJ, et al. Deep neuromuscular block to optimize surgical space conditions during laparoscopic surgery: a systematic review and meta-analysis. Br J Anaesth. 2017;118: 834–842. doi: 10.1093/bja/aex116 28575335

[21] Özdemir-van Brunschot DMD, Braat AE, van der Jagt MFP, Scheffer GJ, Martini CH, Langenhuijsen JF, et al. Deep neuromuscular blockade improves surgical conditions during low-pressure pneumoperitoneum laparoscopic donor nephrectomy. Surg Endosc. 2018;32: 245–251. doi: 10.1007/s00464-017-5670-2 28643056 PMC5770501

[22] Barrio J, Errando CL, García-Ramón J, Sellés R, San Miguel G, Gallego J. Influence of depth of neuromuscular blockade on surgical conditions during low-pressure pneumoperitoneum laparoscopic cholecystectomy: A randomized blinded study. J Clin Anesth. 2017;42: 26–30. doi: 10.1016/j.jclinane.2017.08.005 28803124

[23] Samsa G, Samsa L. A Guide to Reproducibility in Preclinical Research. Academic Medicine. Acad Med; 2019. pp. 47–52. doi: 10.1097/ACM.0000000000002351 29995667 PMC6314499

[24] Samuel S, König-Ries B. Understanding experiments and research practices for reproducibility: An exploratory study. PeerJ. 2021;9. doi: 10.7717/peerj.11140 33976964 PMC8067906

[25] Vlot J, Wijnen R, Stolker RJ, Bax K. Optimizing working space in porcine laparoscopy: CT measurement of the effects of intra-abdominal pressure. Surg Endosc. 2013;27: 1668–1673. doi: 10.1007/s00464-012-2654-0 23239305

[26] Martinoni EP, Pfister CA, Stadler KS, Schumacher PM, Leibundgut D, Bouillon T, et al. Model‐based control of mechanical ventilation: design and clinical validation. Br J Anaesth. 2004;92: 800–807. doi: 10.1093/bja/aeh145 15096447

[27] Tehrani FT, Rogers M, Lo T, Malinowski T, Afuwape S, Lum M, et al. A dual closed-loop control system for mechanical ventilation. J Clin Monit Comput. 2004;18: 111–129. doi: 10.1023/b:jocm.0000032744.99885.38 15362273

[28] Platen P Von, Pomprapa A, Lachmann B, Leonhardt S. The dawn of physiological closed-loop ventilation—A review. Critical Care. Crit Care; 2020. doi: 10.1186/s13054-020-2810-1 32223754 PMC7104522

[29] Ghita M, Neckebroek M, Muresan C, Copot D. Closed-loop control of anesthesia: Survey on actual trends, challenges and perspectives. IEEE Access. 2020;8: 206264–206279. doi: 10.1109/ACCESS.2020.3037725

[30] Judge EP, Hughes JML, Egan JJ, Maguire M, Molloy EL, O’Dea S. Anatomy and bronchoscopy of the porcine lung: A model for translational respiratory medicine. American Journal of Respiratory Cell and Molecular Biology. Am J Respir Cell Mol Biol; 2014. pp. 334–343. doi: 10.1165/rcmb.2013-0453TR 24828366

[31] Kobayashi E, Hishikawa S, Teratani T, Lefor AT. The pig as a model for translational research: Overview of porcine animal models at Jichi Medical University. Transplantation Research. BioMed Central; 2012. p. 8. doi: 10.1186/2047-1440-1-8 23369409 PMC3560993

[32] Szmuk P, Steiner JW, Olomu PN, Ploski RP, Sessler DI, Ezri T. Oxygen reserve index a novel noninvasive measure of oxygen reserve-a pilot study. Anesthesiology. 2016;124: 779–784. doi: 10.1097/ALN.0000000000001009 26978143

[33] NYARWAYA J ‐B MAZOIT J X, SAMII K. Are pulse oximetry and end‐tidal carbon dioxide tension monitoring reliable during laparoscopic surgery? Anaesthesia. 1994;49: 775–778. doi: 10.1111/j.1365-2044.1994.tb04449.x 7978132

[34] Nahas K, Baneux P, Detweiler D. Electrocardiographic monitoring in the Göttingen minipig. Comp Med. 2002;52: 258–264. 12102572

[35] Atkinson TM, Giraud GD, Togioka BM, Jones DB, Cigarroa JE. Cardiovascular and Ventilatory Consequences of Laparoscopic Surgery. Circulation. 2017;135: 700–710. doi: 10.1161/CIRCULATIONAHA.116.023262 28193800

[36] Sodha S, Nazarian S, Adshead JM, Vasdev N, Mohan-S G. Effect of pneumoperitoneum on renal function and physiology in patients undergoing robotic renal surgery. Current Urology. Wolters Kluwer Health; 2015. pp. 1–4. doi: 10.1159/000442842 26989363 PMC4789951

[37] Wever KE, Bruintjes MHD, Warlé MC, Hooijmans CR. Renal perfusion and function during pneumoperitoneum: A systematic review and meta-analysis of animal studies. PLoS One. 2016;11. doi: 10.1371/journal.pone.0163419 27657740 PMC5033590

[38] Wysocki M, Brunner JX. Closed-Loop Ventilation: An Emerging Standard of Care? Critical Care Clinics. Crit Care Clin; 2007. pp. 223–240. doi: 10.1016/j.ccc.2006.12.011 17368167

[39] Sturrock S, Williams E, Dassios T, Greenough A. Closed loop automated oxygen control in neonates—A review. Acta Paediatrica, International Journal of Paediatrics. Acta Paediatr; 2020. pp. 914–922. doi: 10.1111/apa.15089 31715041

[40] Hatipoglu S, Akbulut S, Hatipoglu F, Abdullayev R. Effect of laparoscopic abdominal surgery on splanchnic circulation: Historical developments. World J Gastroenterol. 2014;20: 18165. doi: 10.3748/wjg.v20.i48.18165 25561784 PMC4277954

[41] Balick-Weber CC, Nicolas P, Hedreville-Montout M, Blanchet P, Stéphan F. Respiratory and haemodynamic effects of volume-controlled vs pressure-controlled ventilation during laparoscopy: a cross-over study with echocardiographic assessment. Br J Anaesth. 2007;99: 429–435. doi: 10.1093/bja/aem166 17626027

[42] Diaz-Cambronero O, Flor Lorente B, Mazzinari G, Vila Montañes M, García Gregorio N, Robles Hernandez D, et al. A multifaceted individualized pneumoperitoneum strategy for laparoscopic colorectal surgery: a multicenter observational feasibility study. Surg Endosc. 2019;33: 252–260. doi: 10.1007/s00464-018-6305-y 29951750

[43] Rocco PRM, Marini JJ. What have we learned from animal models of ventilator-induced lung injury? Intensive Care Medicine. Nature Publishing Group; 2020. pp. 2377–2380. doi: 10.1007/s00134-020-06143-x PMC7270159 32500178

